# Supplementary material for: State of the art in selection of variables and functional forms in multivariable analysis—outstanding issues
Source: Diagn Progn Res. 2020 Apr 2;4:3. doi: 10.1186/s41512-020-00074-3 (PMC7114804; doi:10.1186/s41512-020-00074-3)
Supplement: Supplementary file 1 — Additional file 1. Web supplement. [file 41512_2020_74_MOESM1_ESM.docx]

# Web supplement

1. 1. Methods based on spline functions

Splines (piecewise polynomial functions) come in many shapes and forms. A basic dichotomy is between regression splines, which focus on the polynomial choice and finding a set of knot locations, and smoothing splines, which focus on minimization of a penalty function.

Regression splines are particularly attractive due to their simplicity, ability to be included in any regression software and relatively simple mathematical form. Estimation of confidence intervals does not present a particular challenge and can be done with standard regression theory. Although there exist several different types of spline basis functions (including truncated polynomials, B-splines, cubic splines, natural splines), in practice the type of basis chosen should not dramatically alter the fitted function. The choice of basis depends on numerical stability and interpretability. Two preferred approaches are B-splines, which are numerically stable, and natural cubic splines, which are more flexible and constrained to linearity in the tails.

With both B-splines and natural splines the number and placement of knots must be specified. Allowing a model to treat knot positions as parameters may increase flexibility but incur a heavy computational cost and instability. Automatic knot selection has also been suggested, typically based on stepwise procedures [20]; however, this approach is computationally intensive and hard to implement in practice. Stone [18] has shown that provided there are enough knots to supply the required flexibility, the precise placement of knots is not crucial. Harrell [5] suggests the use of four knots at appropriate quantiles for moderate samples sizes, three knots for smaller datasets (<30 observations) and five knots for larger datasets. Ruppert et al. [12] emphasise the benefit of unequally spaced knots, to avoid placement in empty regions of the domain of the continuous variable.

Smoothing splines extend cubic splines by placing a knot at each observation and adding a roughness penalty to control the smoothness of the fit. Eilers and Marx [3] introduced P-splines by extending and simplifying ideas first seen in O’Sullivan [6]. Eilers and Marx’s approach has been implemented in most statistical software packages. P-splines utilise B-splines with a large number of equidistant knots which are controlled by a discrete penalty specified in terms of differencing operators. More recently, Eilers and Marx [4], describing their experience of using P-splines, strongly recommended equally-spaced knots.

Thin-plate regression splines have some very desirable properties. Based on the work by Duchon [2], Wood [22] showed how to restrict thin-plate splines and provided a computationally efficient method. With Thin-plate regression splines, the analyst does not need to specify the number or placement of knots, nor to select a basis function. The method has become more widespread with the introduction of mgcv, a well-written and documented R package that allows for automatic penalty optimization using generalised cross validation, and an informative book on generalised additive models using R [23]. For penalised splines, estimation of confidence intervals requires approximate Bayesian methods [17, 19]. In a later work Wood [23] illustrated how to improve performance of such intervals.

Although the mathematical properties of splines are well-understood, thorough comparison of methods is sparsely reported in the statistical literature. Ruppert et al. [13]( described their experience of semiparametric regression in the years 2003-2007, Welham et al. [21] compared smoothing splines, P-splines and penalised splines using a truncated power function basis, and Binder et al. [1] compared splines with fractional polynomials. STRATOS recognises the need for further in-depth review papers and systematic comparisons among methods. Furthermore, guidance is needed on how to present results of a regression model with splines. Common practice would be a visual representation of the spline function along with 95% confidence bands. However, in many cases clinicians would be more interested in a simpler numerical representation such as by reporting contrasts between typical values of the continuous variable, or highlighting points of changing behaviour in the fit. A review of spline procedures has been published by members of TG2 that illustrates the use of splines using R.

1. 2. Fractional polynomials

With fractional polynomials (FPs), the aim is to extract full information from continuous variables in univariable and multivariable settings, resulting in models with simple and plausible functional forms. The selected model and functional forms should be interpretable from a subject-matter perspective. Interpretability, transportability and general usability of a model demand simplicity. Complex models, including complex functions of continuous covariates, are not useful when the aim is essentially descriptive.

A fractional polynomial is a simple function of a continuous covariate X. Starting from a straight-line model 𝛽_1_Χ, a natural extension is a power transformation model β_1_Χ^p^. Royston and Altman [7] formalised such a model by calling it a first-degree fractional polynomial or FP1 function, where the power p is chosen from a restricted set S ={−2,−1,−0.5, 0, 0.5, 1, 2, 3}, with a power of 0 defining the log transformation of X. Extension of FP1 functions to the more complex and flexible two-term FP2 functions follows by defining FP2 functions with powers (p_1_, p_2_) as 𝛽_1_X^p1^ + 𝛽_2_ X^p2^, where p_1_ and p_2_ are taken from S. If p_1_ =p_2_=p the FP2 class is defined as 𝛽_1_X_p_ + 𝛽_2_ X_p_ log X, a so-called repeated-powers FP2 model. FP1 functions are monotonic and those with power p<0 have an asymptote as X→∞. FP2 functions may be monotonic or unimodal (i.e., have one maximum or one minimum for some positive values of X), and they have an asymptote as X→∞ when both p_1_ and p_2_ are negative. Generalization to FPm (m > 2) functions is straightforward, but the FP2 class is complex enough in many applications in the health sciences. The class of FP1 and FP2 functions is small (8 FP1 functions, 36 FP2 functions) but it includes a large variety of different functional forms. In contrast to splines, FPs are functions defined globally and cannot identify or respond to local features of the data to hand. For further details, see Royston and Sauerbrei [11] or the website <http://mfp.imbi.uni-freiburg.de/>.

# Function selection procedure

Royston and Sauerbrei. [11] defined a function selection procedure (FSP) based on a closed test procedure. The complexity of the finally chosen function is predicated on preliminary decisions as to the nominal significance level (𝛼) and the degree (m) of the most complex FP model allowed. Typical choices are 𝛼 =0.05 and m = 2 (FP2). With FP2 as the most complex allowed FP function, FSP selects an FP function according to the following procedure:

1. Test the best-fitting FP2 model for X at significance level 𝛼 against the null model using 4 d.f. If the test is not significant, stop, concluding that the effect of X is “not significant” at the 𝛼 level. Otherwise continue.

2. Test the best-fitting FP2 for X against a straight line (no transformation of X) at the 𝛼 level using 3 d.f. If the test is not significant, stop, the final model including untransformed X. Otherwise continue.

3. Test the best-fitting FP2 for X against the best FP1 for X at the 𝛼 level using 2 d.f. If the test is not significant, the final model uses an FP1 transformation of X, otherwise the final model uses FP2. End of procedure.

The test at step 1 is of overall association of the outcome with X. The test at step 2 examines the evidence for nonlinearity. The test at step 3 chooses between a simpler or more complex nonlinear model. The test at step 1 is not needed in a univariate analysis.

The multivariable fractional polynomial (MFP) procedure combines [variable selection with backward elimination](http://mfp.imbi.uni-freiburg.de/varsel) and selection of an FP function to model non-linearity (<http://mfp.imbi.uni-freiburg.de/mfp>). For both parts, nominal significance level are the key parameters and their choice has a strong influence on the MFP model selected. A more detailed discussion of issues in practice and some comparisons of FP and spline based functions can be found in the literature [11, 16].

# Presentation, stability and inference

In general, several models have a similar fit and therefore a similar functional form, despite differences among the powers chosen. Therefore, parameter estimates and powers are not interpretable *per se*, but taken together they describe a function which estimates the functional influence of a variable on the outcome. It is strongly recommended to give a graphical presentation of the functional influence and in addition it can be helpful to present the functional influence in a tabular way [8, 11].

Influential points may have a severe influence on the selection of the specific function. To cope with this issue and to improve robustness of fractional polynomials functions, preliminary transformations may be used [10, 11].

Estimating regression parameters and corresponding variances for a derived model ignores the function selection process and are conducted as if the power terms would have been pre-specified. Consequently, parameter estimates are biased and confidence intervals are too narrow. It is proposed to use shrinkage to receive more realistic parameter estimates and the bootstrap to get a more realistic estimate of the variance of a function [11, 14, 15].

Specifically in the multivariable context bootstrap investigations are recommended as they can provide important insights into the selected model and its stability. Such investigations can show that several alternative models may explain the data, but the sample size was too small for detecting the influence of a variable or that a non-linear function would fit the data better [9, 10, 14, 15].

References for Web supplement

1. Binder H, Sauerbrei W, Royston P. Comparison between splines and fractional polynomials for multivariable model building with continuous covariates: a simulation study with continuous response. Statistics in Medicine. 2013;32:2262–2277.
2. Duchon J. Splines minimizing rotation-invariant semi-norms in Solobev spaces. In Construction Theory of Functions of Several Variables. Berlin: Springer; 1977.
3. Eilers PHC, Marx BD. Flexible smoothing with B-splines and penalties (with comments and rejoinder). Statistical Science. 1996;11:89-121.
4. Eilers PHC, Marx BD. Splines, knots, and penalties. Wiley Interdisciplinary Reviews. Computational Statistics (Print). 2010;2:637–653.
5. Harrell FE. Regression Modeling Strategies. Online course material. <http://biostat.mc.vanderbilt.edu/tmp/course.pdf.2017;> Accessed 29 November 2018.
6. O'Sullivan F. A statistical perspective on ill-posed inverse problems. Statistical science. 1986;1:502-518.
7. Royston P, Altman DG. Regression using fractional polynomials of continuous covariates: parsimonious parametric modelling. Applied Statistics. 1994;43:429–467.
8. Royston P, Ambler G, Sauerbrei W. ‘The use of fractional polynomials to model continuous risk variables in epidemiology’ International Journal of Epidemiology. 1999;28:964-974
9. Royston P, Sauerbrei W. ’Stability of multivariable fractional polynomial models with selection of variables and transformations: a bootstrap investigation.’ Statistics in Medicine. 2003;22:639-659
10. Royston P, Sauerbrei W. Improving the robustness of fractional polynomial models by preliminary covariate transformation: a pragmatic approach. Computational Statistics and Data Analysis.2007;51: 4240-4253.
11. Royston P, Sauerbrei W. Multivariable Model-building. A Pragmatic Approach to Regression Analysis Based on Fractional Polynomials for Continuous Variables. Wiley, Chichester; 2008.
12. Ruppert D, Wand MP, Carroll RJ. Semiparametric regression during 2003-2007. Electronic Journal of Statistics. 2009;3:1193-1256.
13. Ruppert D, Wand MP, Carroll RJ. Semiparametric Regression. Cambridge University Press. Cambridge; 2003.
14. Sauerbrei W, Royston P. Building multivariable prognostic and diagnostic models: Transformation of the predictors by using fractional polynomials. Journal of the Royal Statistical Society. 1999;162:71-94
15. Sauerbrei W, Royston P. Modelling to extract more information from clinical trials data : on some roles for the bootstrap. Statistics in Medicine. 2007;26: 4989-5001.
16. Sauerbrei W, Royston P, Binder H. Selection of important variables and determination of functional form for continuous predictors in multivariable model building. Statistics in Medicine. 2007;6: 5512-5528
17. Silverman BW. Some aspects of the spline smoothing approach to non-parametric regression curve fitting. Journal of the Royal Statistical Society: Series B (Methodological). 1985;47(1):1-21.
18. Stone CJ, Koo CY. Additive splines in statistics. Proceedings of the American Statistical Association. 1985;45-48.
19. Wahba G. Bayesian “confidence intervals” for the cross-validated smoothing spline. Journal of the Royal Statistical Society: Series B (Methodological). 1983;45(1):133-50.
20. Wand MP. A comparison of regression spline smoothing procedures. Computational Statistics. 2000;15:443-462.
21. Welham SJ, Cullis BR, Kenward MG, Thompson R. A comparison of mixed model splines for curve fitting. Australian & New Zealand journal of statistics. 2007;49:1-23.
22. Wood S. Generalized Additive Models. Chapman & Hall/CRC, New York; 2006.
23. Wood S. Thin plate regression splines. Journal of the Royal Statistical Society, Series B. 2003;65:95-114.
24. Wood S. On confidence intervals for generalised additive models based on penalized regression splines. Australian and New Zealand Journal of Statistics. 2006; 48:445-464
